# The Increased Expression of CCL20 and CCR6 in Rectal Mucosa Correlated to Severe Inflammation in Pediatric Ulcerative Colitis

**DOI:** 10.1155/2015/856532

**Published:** 2015-01-27

**Authors:** Keiichi Uchida, Yuhki Koike, Kiyoshi Hashimoto, Susumu Saigusa, Mikihiro Inoue, Kohei Otake, Koji Tanaka, Kohei Matsushita, Yoshiki Okita, Hiroyuki Fujikawa, Toshimitsu Araki, Yasuhiko Mohri, Masato Kusunoki

**Affiliations:** Department of Gastrointestinal and Pediatric Surgery, Mie University Graduate School of Medicine, 2-174 Edobashi, Tsu, Mie 514-8507, Japan

## Abstract

*Background/Aims*. The aim of this study is to clarify the differences of CCL20 and CCR6 expression, chemokine correlated to intestinal homeostasis, between pediatric and adult ulcerative colitis (UC) patients. *Methods*. Onehundred forty-one patients who underwent proctocolectomy were divided to two groups including childhood-onset UC (CUC, <16 years old, *n* = 24) and adult-onset UC (AUC, ≧16 years old, *n* = 117). A total of 141 formalin-fixed, paraffin-embedded tissue samples of rectum were obtained from these patients. Histological inflammation of rectum in resected specimen was evaluated by using Geboes histological assessment. In immunohistochemistry study, the CCL20 expression was evaluated by intensity and the stained area, and the CCR6 expression was evaluated by lymphocytes infiltration pattern. *Results*. CCL20 score and CCR6 positive lymphocytes infiltration pattern were statistically significantly correlated with histological inflammation severity of UC in all patients (*P* < 0.05). CCL20 and CCR6 expression in CUC were statistically significantly higher than that in AUC in all or pathologically severe cases (*P* < 0.05). *Conclusions*. CCL20 and CCR6 may play a significant role in local damage and pathological changes in UC especially pediatric patients. In the future, our understanding of the differences in CCL-CCR6 interaction between adults and children may lead to the pathogenesis of IBD.

## 1. Introduction

Ulcerative colitis (UC) in children is clinically characterized of being more extensive, severe, and progressive compared to UC in adults; however, the reason remains unclear [1–3]. Several studies addressing the pathogenesis of inflammatory bowel disease (IBD) have focused on impaired immune response through mucosal cytokine or chemokine production; however, the pathogenesis also has not resolved yet. It is of no doubt that the disruption of intestinal homeostasis can lead to IBD characterized by self-destructive intestinal immunity. Intestinal homeostasis is regulated by lymphoid tissue genesis, which is induced by commensal bacteria through the different pattern recognition receptors (PRRs) in intestinal epithelial cell (IEC) signaling [4]. Microbial recognition by IECs leads to the production of different effector molecules that are secreted either into the lumen, such as antimicrobial peptides such as cryptidins and beta-defensin 3, or at the basolateral side to establish a direct communication with cells of stroma and the immune system. These signaling cascades involve nucleotide-binding oligomerization domain containing (NOD) 1 innate receptor in epithelial cells to produce beta-defensin 3 and CCL20 to induce cryptopatch maturation assisted by toll-like receptors (TLRs) and NOD2, and TLR-mediated production of CCL20, and recruitment of B cell to lymphoid tissues [4].

In IBD, leukocyte infiltration into the intestine is fundamental event in disease development and progression where the chemokine and their receptors are orchestrating the trafficking of leukocytes [5]. CCL20 is predominantly expressed in the inflamed intestinal epithelium and plays an important role in lymphocyte and dendritic cell activation and recruitment to the colonic epithelia [6–8]. Previous studies demonstrated that CCL20 expression levels in the colonic epithelia of patients with IBD were higher than in normal colonic epithelia [9, 10]. CCR6 is expressed in most B cells and subsets of T cells, immature dendritic cells, activated neutrophils, and lymphoid tissues, including the lymph node, spleen, and the gut mucosal immune system [7, 11]. Varona et al. [12] conducted an in vivo study demonstrating that CCR6 is attracted to the colonic mucosal layer and plays a crucial role in the development of IBD.

In the current study, we investigated CCL20 expression in the colonic mucosa and its receptor CCR6 expression of the inflammatory cells in the rectal mucosal layer of surgically resected specimen and clarify the significance of differences between CUC and adult-onset AUC.

## 2. Methods

### 2.1. Patients and Samples

We experienced 141 patients with UC who underwent proctocolectomy from 2003 to 2011 in Mie University Hospital. Patients were divided to two groups including childhood-onset group (CUC, <16 years old, *n* = 24) and adult-onset group (AUC, ≧16 years old, *n* = 117). A total of 141 formalin-fixed, paraffin-embedded (FFPE) tissue samples of rectum were obtained from these patients. The study design was approved by the ethics review board of Mie University Hospital. All patients or guardians provided written informed consent to allow the collection and use of their tissues for this study.

The diagnosis of UC was based on clinical features, laboratory test results indicating inflammation, and endoscopic and histopathological findings. Disease severity was defined according to the diagnostic criteria for UC severity determination established by the Research Committee of IBD of the Ministry of Health and Welfare in Japan in 1994 [13].

All resected specimens obtained after restorative proctocolectomy were routinely fixed in formalin, stained with hematoxylin and eosin, and evaluated microscopically. Histological inflammation of rectum of resected specimen was evaluated by using Geboes histological assessment for ulcerative colitis [14]. Samples with grade 0 or 1 inflammation were classified as mild inflammation levels, samples with grade 2 or 3 inflammation were classified as moderate inflammation levels, and samples with grade 4 or 5 inflammation were classified as severe inflammation levels.

### 2.2. Immunohistochemistry

FFPE specimens of rectum were sliced in 2 *μ*m thick sections. After deparaffinization and dehydration, the sections were incubated in 10 mM sodium citrate buffer (pH 6.0) and autoclaved at 121°C for 10 min for antigen retrieval. Following an additional incubation in 3% hydrogen peroxide for 10 min, sections were then blocked and incubated with primary antibody overnight at 4°C. Human CCL20/MIP-3*α* antibodies (monoclonal mouse IgG1 clone #67310, R&D Systems, USA; dilution: 1 : 250) and human CCR6 antibodies (monoclonal mouse IgG2B clone #53103, R&D Systems; dilution: 1 : 50) were used as primary antibodies for implementation of the labeled streptavidin-biotin method (EnvisionTM+Dual Link System-HRP, Dako Cytomation, Denmark) and 3,3′-diaminobenzidine (Dako Cytomation, Denmark) staining. All sections were counterstained with hematoxylin and were dehydrated and mounted. We stained at least 3 sections per specimen to confirm reproducibility. Negative controls were run simultaneously with preimmune immunoglobulin.

### 2.3. Immunohistochemical Evaluation

**Figure 1:**
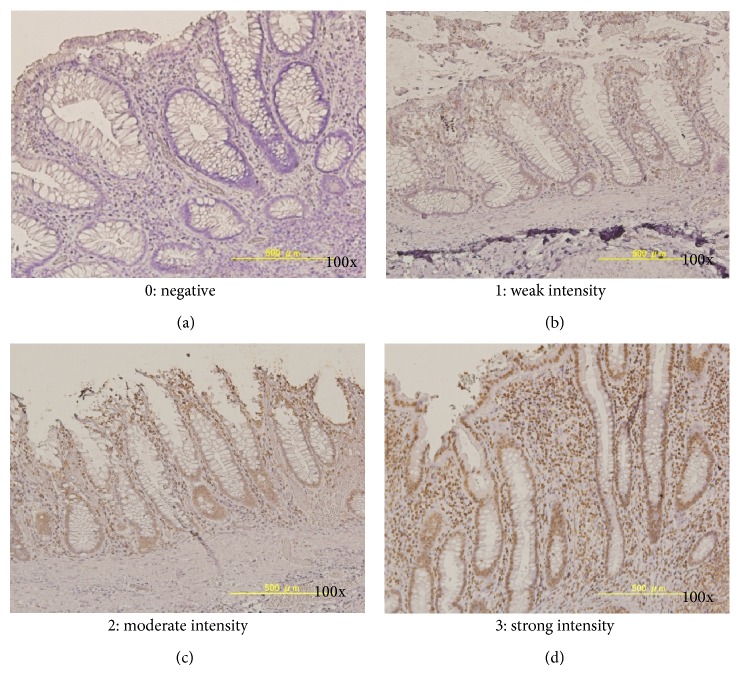
Immunohistochemical findings for CCL20 in the rectal mucosa. CCL20 expression was observed in the nucleus of epithelial cells, inflammatory cells, and lymphoid follicles. Different grades of CCL20 intensity in epithelial cells were evaluated as follows: grade 0 (negative, (a)), 44 specimens; grade 1 (weak, (b)), 37 specimens; grade 2 (moderate, (c)), 48 specimens, and grade 3 (severe, (d)), 12 specimens, respectively. Original magnification, 100x.

Sections were observed under a light microscope. CCL20 were expressed in the intestinal epithelial cells in the propria mucosa. The staining intensities of epithelial cells were graded as follows: grade 0 (negative), no staining of epithelial cells; grade 1 (weak intensity), 10% to 30% positive epithelial cells; grade 2 (moderate intensity), 30% to 50% positive epithelial cells; and grade 3 (strong intensity), greater than 50% positive epithelial cells (Figure 1). We also evaluated the stained area of CCL20 expression in the epithelium. The stained area was graded by positive stained epithelial cell numbers (all epithelial cell numbers at the counted same area under a light microscope) as follows: grade 1, ≦0.33; grade 2, 0.33< and ≦0.66; and grade 3, 0.66<. The CCL immunohistochemical (IHC) scores were calculated by multiplying intensity score by the stained area score, ranging from 0 to 9.

CCR6 expression was predominantly observed in the plasma membrane of the infiltrating inflammatory cells. We evaluated the infiltrating pattern of CCR6-positive cells, graded as follows: grade 0, no staining of infiltrating cells; grade 1, focal (only surface of mucosa); and grade 2, diffuse (surface to bottom of mucosa).

### 2.4. Statistical Analysis

All statistical analyses were performed using Stat View 5.0 for Windows (SAS Institute Inc., Cary, NC, USA). Contingency tables were analyzed using Fisher's exact probability test or chi-squared test with Yates's correction. Correlations between continuous variables and categorical variables were evaluated using the Mann-Whitney *U* test for 2 groups and Kruskal-Wallis test for more than 3 groups. A *P* value of less than 0.05 was considered to be statistically significant.

## 3. Results

### 3.1. Patient Demographics and Disease Characteristics

Patients' characteristics are shown in Table 1. The gender ratio (male/female) was 2.3 (17/7) in CUC and 1.5 (70/47) in AUC and had no significant difference. The median age at UC diagnosis was 13 years (range: 1–15 years) in CUC and 29 years (range: 16–82 years) in AUC. The median duration of disease at operation was 3 years (range: 0.3–28 years) in CUC and 5 years (range: 0.1–28 years) in AUC. There is no difference in clinical severity, extent of disease, and histological inflammation at operation between two groups.

### 3.2. Immunohistochemical Findings and Evaluation of CCL20 and CCR6 Expression

CCL20 expression was observed in the nucleus of epithelial cells, inflammatory cells, and lymphoid follicles (Figure 1). Different grades of CCL20 intensities in epithelial cells were evaluated as follows: grade 0, 44 specimens; grade 1, 37 specimens; grade 2, 48 specimens; and grade 3, 12 specimens.

**Figure 2:**
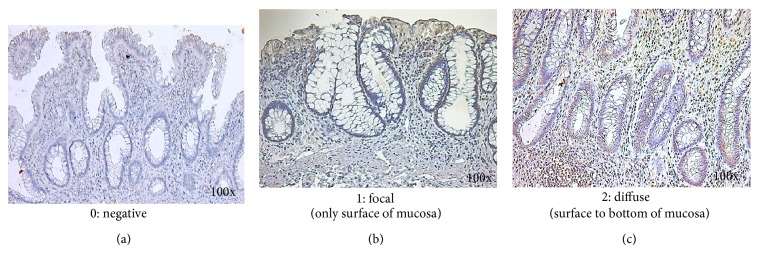
Immunohistochemical findings for CCR6 in the rectal mucosa. CCR6 expression was observed in the nucleus or cytoplasm of epithelial cells, infiltrating inflammatory cells, and endothelial cells. Different grades of CC6 infiltrating pattern in epithelia were evaluated as follows: grade 0 (negative, (a)), 38 specimens; grade 1 (focal, (b)), 59 specimens; grade 2 (diffuse, (c)), 44 specimens, respectively. Original magnification, 100x.

CCR6 expression was predominantly observed in the plasma membrane of infiltrating inflammatory cells (Figure 2). Different grades of CCR6 infiltrating pattern in mucosal layer were evaluated as follows: grade 0 (negative), 38 specimens; grade 1 (focal), 59 specimens; and grade 2 (diffuse), 44 specimens, respectively.

Table 2 demonstrated the relationship between pathological inflammation severity and CCL20 and CCR6 expression in all patients. CCL20 IHC scores (mean ± SD) were 1.65 ± 0.32 in mild severity, 2.43 ± 0.38 in moderate severity, and 3.18 ± 0.38 in severe severity, respectively. CCR6 scores (mean ± SD) were 0.92 ± 0.68 in mild severity, 1.14 ± 0.84 in moderate severity, and 1.34 ± 0.72 in severe severity, respectively. CCL20 IHC score and CCR6 positive inflammation infiltration pattern had statistically significant difference between mild and severe histological inflammation in rectum (*P* < 0.05).

Table 3 demonstrated the comparison of CCL20 and CCR6 expression between CUC and AUC. CCL20 IHC score in COG was 3.07 ± 0.47 compared with 2.43 ± 0.38 in AUC, and CCR6 score in CUC was 1.52 ± 0.67 compared with 1.06 ± 0.77 in AUC. Both scores in CUC were statistically significant higher than that in AUC (*P* < 0.05). Moreover, in pathologically severe cases, CCL20 IHC score in CUC was 4.13 ± 3.52 compared with 2.75 ± 2.91 in AUC, and CCR6 score in CUC was 1.64 ± 0.77 compared with 1.21 ± 0.49 in AUC. Both scores in CUC were statistically significant higher than that in AUC (*P* < 0.05).

## 4. Discussion

CUC is characterized by extensive intestinal involvement and rapid early progression. Jakobsen et al. [1] demonstrated that CUC patients had more extensive disease, were more often treated with systemic steroids and azathioprine (AZA), and had a higher frequency of steroid dependency and a more severe disease course compared to adult UC patients. Van Limbergen et al. [2] reported that 82% of CUC was extensive at diagnosis, versus 48% of AUC; 46% of CUC progressed to develop extensive colitis during follow-up. The median time to first surgery was shorter in CUC than AUC. However, why CUC is relatively more severe compared with AUC is not described precisely in the literature.

It is hard to perform the comparative research between adult and children maybe because of the paucity of hospitals to treat adult and pediatric IBD patients in the same unit in the world. It is characteristic that both AUC and CUC were surgically treated by same surgical team, same surgical procedure, and same perioperative management at our institute. So, we think that our report is very valuable to compare pediatric and adult surgical patients with UC.

In this study, we evaluated CCL20 production by the intensity and area in colonic surface cells and CCR6 production by interstitial infiltration pattern of inflammatory cells in colonic mucosal layer. Increased enterocyte CCL20 production plays an important role in lymphocyte activation and recruitment to the colonic epithelium in IBD [9]. And CCR6 expression was observed in the infiltrating inflammatory cells in mucosal layer. Infiltration appearance divided to 2 different patterns: focal (only surface of mucosa) and diffuse (surface to bottom of mucosa).

We demonstrated that CCL20 and CCR6 expression are correlated to the histological severity in rectum resected in all CUC and AUC cases. And, in severe cases, there is statistically significant difference in CCL20 expression between CUC and AUC. CCL20 expression in CUC is higher than that in AUC in same level severity in pathological examination.

IBD is a group of autoimmune diseases characterized by nonspecific inflammation in the gastrointestinal tract. Recent investigations suggest that activation of Th17 cells and/or deficiency of regulatory T cells (Treg), as these are two major types of CCL20-responsive cell types, is involved in the pathogenesis of IBD [4, 5, 15]. Ghadjar et al. [15] demonstrated increased circulating IL-17 and Treg cells and a decreased suppressive function of Treg lymphocytes in peripheral blood of patients with IBD. In vitro, Th17 cells responding to CCL20 promote migration of Th17 and Treg cells in a CCR6-dependent manner [8]. Moreover, Th17 cells, by producing CCL20, could also attract other Th17 cells via CCR6 [16, 17]. CCR6 is important for Th17 migration to inflammatory tissues and may mediate an amplifying regulation to sustain inflammatory response. Lack of CCR6 in Th17 cells reduces the severity of experimental autoimmune encephalomyelitis and Th17 and Treg recruitment into inflammatory tissues [8]. Similarly, CCR6 on Treg cells is also important for their recruitment into inflammatory tissues. CCL20-CCR6 interaction, by recruitment of Treg cells, may initiate a feedback anti-inflammatory response in autoimmune diseases including IBD. In the future, our understanding of the functional differences in Th17 and Treg cells via CCR6 between adults and children may lead to the pathogenesis of IBD.

The ligand-receptor pair CCL20-CCR6 is responsible for the chemoattraction of immature dendritic cells (DC), effector/memory T cells, and B cells and plays a role at mucosal surfaces under homeostatic and inflammatory conditions, as well as in pathology, including IBD [6]. CCL20 expression by the intestinal epithelium is observed in the epithelium of cytokine-stimulated or bacteria-infected human intestinal xenografts and in the epithelium of inflamed human colon [8]. The expression of the CCL20 protein in colonic mucosa was detected in UC patients and experimental dextran sulfate sodium- (DSS-) induced experimental colitis in mice in several studies [18]. The blockade of CCL20-CCR6 interaction by an anti-mouse CCL20 monoclonal antibody (MAb) or the desensitization of CCR6 by pretreatment with CCL20 significantly attenuated the infiltration of both T cells and B cells into the colitic legions [19]. Moreover, neutralization of CCL20 expression using its monoclonal antibody reduced 2,4,6-trinitrobenzene sulfonic acid- (TNBS-) mediated colonic injury and T cell recruitment [20]. It was reported that CCL20 expression in blood mononuclear cells is also associated with disease severity in pretreated and altered inflammatory responses in patients with UC [21].

Our previous study investigated whether the expression of CCL20 and CCR6 was correlated with the development of UC-associated neoplasia in adult UC patients [22]. The results suggest that an evaluation of CCL20 expression in the rectal mucosa may be useful to identify patients who are at a high risk for developing UC-associated neoplasia in adult. Further research in pediatric IBD may clarify the pathogenesis of IBD, resolve the mechanism of chronic and recurrent inflammation, and finally eradicate these life-long refractory diseases. CCL20 and CCR6 may play a significant role in local damage and pathological changes in UC especially pediatric patients. In the future, our understanding of the differences in CCL20-CCR6 interaction between adults and children may lead to the pathogenesis of IBD.

## Figures and Tables

**Table 1:** Patients characteristics.

	CUC (*n* = 24)	AUC (*n* = 117)	*P* value
Gender (male/female)	17/7	70/47	n.s.
Age at UC diagnosis, years (median, range)	13.0 (1–15)	29.0 (16–82)	*P* < 0.05
Duration of disease before surgery, years (median, range)	3 (0.3–28)	5 (0.1–28)	n.s.
Clinical severity at operation, *n*			
Mild	10 (41.7%)	51 (43.6%)	n.s.
Moderate	10 (41.7%)	50 (42.7%)	
Severe	4 (17.6%)	16 (14.3%)	
Extent of disease at operation, *n*			
Pancolitis	21 (87.5%)	95 (81.2%)	n.s.
Left-sided colitis	3 (12.5%)	22 (18.8%)	
Pathological Inflammation in rectum, *n*			
Mild (grades 0, 1)	6 (14.0%)	10 (10.2%)	n.s.
Moderate (grades 2, 3)	11 (25.6%)	34 (34.7%)	
Severe (grades 4, 5)	26 (60.4%)	44 (45.1%)	

CUC; childhood-onset; AUC; adult-onset.

Correlations were evaluated using Fisher's exact probability test or chi-squared test, the Mann-Whitney *U* test, and Kruskal-Wallis test. A *P* value of less than 0.05 was considered to be statistically significant.

**Table 2:** The relationship between pathological inflammation severity and CCL20 and CCR6 expression.

	Mild	Moderate	Severe	*P* value
CCL20 IHC score	1.65 ± 0.32^*^	2.43 ± 0.38^**^	3.18 ± 0.38^∗,∗∗^	^∗,∗∗^ *P* < 0.05

CCR6 score	0.92 ± 0.68^*^	1.14 ± 0.84	1.34 ± 0.72^*^	^*^ *P* < 0.05

Correlations were evaluated using the Mann-Whitney *U* test. A *P* value of less than 0.05 was considered to be statistically significant.

In upper area of Table 2, ∗: demonstrated the comparison between mild and severe group and ∗∗: demonstrated the comparison between moderate and severe group in CCL20 score (^∗,∗∗^ *P* < 0.05). In lower area of Table 2, ∗: demonstrated the comparison between mild and severe group in CCR6 (^*^ *P* < 0.05).

**Table 3:** The comparison of CCL20 and CCR6 expression between CUC and AUC.

	CUC	AUC	*P* value
All cases			
CCL20 score	3.07 ± 0.47	2.43 ± 0.38	*P* < 0.05
CCR6 score	1.52 ± 0.67	1.06 ± 0.77	*P* < 0.05
Severe cases			
CCL20 score	4.13 ± 3.52	2.75 ± 2.91	*P* < 0.05
CCR6 score	1.64 ± 0.77	1.21 ± 0.49	*P* < 0.05

CUC; childhood-onset UC, AUC; adult-onset UC.

Correlations were evaluated using the Mann-Whitney *U* test. A *P* value of less than 0.05 was considered to be statistically significant.
